# Value of Active Warming Devices for Intraoperative Hypothermia Prevention—A Meta-Analysis and Cost-Benefit Analysis

**DOI:** 10.3390/ijerph182111360

**Published:** 2021-10-28

**Authors:** He Xu, Zijing Wang, Yijuan Lu, Xin Guan, Yue Ma, Daniel C. Malone, Jack Warren Salmon, Aixia Ma, Wenxi Tang

**Affiliations:** 1 School of International Pharmaceutical Business, China Pharmaceutical University, No. 639 Longmian Street, Nanjing 211198, China; xuhe@stu.cpu.edu.cn (H.X.); 3319040839@stu.cpu.edu.cn (Z.W.); 1347402@stu.cpu.edu.cn (Y.L.); gg_cpu@163.com (X.G.); 17302512206@163.com (Y.M.); aixiama73@126.com (A.M.); 2 Center for Pharmacoeconomics and Outcomes Research, China Pharmaceutical University, No. 639 Longmian Street, Nanjing 211198, China; 3 College of Pharmacy, University of Utah, Salt Lake City, UT 84101, USA; malone@pharmacy.arizona.edu; 4 College of Pharmacy, University of Illinois Chicago, 833 South Wood Street, Chicago, IL 60612, USA; jsjwsalmon@gmail.com

**Keywords:** active warming devices, intraoperative hypothermia, meta-analysis, cost-benefit analysis

## Abstract

Purpose: Historically, studies suggested that intraoperative hypothermia (IH) could result in significant resource consumption, but more recent studies have found the opposite. The purpose of this study is to estimate the value of active warming devices for IH prevention based on synthesized evidence. Methods: A cost-benefit analysis was conducted using the effect of active warming versus passive warming devices for intraoperative hypothermia from a meta-analysis. The item-based aggregated treatment cost approach was adopted to estimate the cost of each adverse event, which was then weighted to calculate the total cost of IH. Results: IH was associated with higher risks of bleeding, surgical site infection, and shivering compared with normothermia. The cost of one case of IH was $363.80, and the use of active warming devices might save $152.80. Extra investment in active warming (e.g., $291.00) might only be cost-beneficial when the minimum willingness-to-pay is $150.00. Conclusions: Synthesized evidence showed that the cost of IH might be overestimated. Furthermore, the value of using active warming devices remains uncertain because the willingness to pay may vary between decision-makers. As not enough awareness of hypothermia prevention in some countries, further research into the clinical use of active warming devices during major surgeries is warranted.

## 1. Introduction

Body temperature is an important vital sign of human beings. Normal body temperature is maintained at approximately 37 °C by neurohumoral. It is essential for maintaining body function, physiological stability, and normal metabolism. However, due to the effects of anesthesia, the duration of surgery, fluid infusion, and other factors, the body’s thermoregulation mechanism is easily impaired, which makes intraoperative hypothermia (IH) (core temperature < 36 °C) comes a common complication during major surgery [1]. IH has been associated with adverse events such as surgical site infection [2], increased intraoperative blood loss [3], pain, and shivering [4]. This may increase the duration of surgery [5], intensive care unit stays [6], hospital stay [7], and reportedly increase the cost of treatment [8]. In recent decades, perioperative body temperature management has been recognized as an important component in the clinical pathway of enhanced recovery after surgery [9].

Currently, body temperature protection methods can be broadly divided into two categories: active warming and passive warming devices. The latter work by covering the patient’s body with substances that have no heat-producing function to reduce heat dissipation through the skin, including covering quilts, insulation blankets, and surgical sheets, etc. However, the effect of this method is very limited. By contrast, active warming is an effective strategy for the prevention of IH, which is based on increasing the total body heat to compensate for the body temperature decrease due to heat dissipation [9]. Active warming systems such as forced-air warming (FAW) and circulating-water warming have been widely used in clinical practice; FAW application in major surgeries is now recommended by guidelines, particularly in obstetrics, gynecology [10,11], orthopedics [12,13], cardiology [14], abdominal surgery [15,16,17], laparoscopic cholecystectomy [18], and prostatectomy [19].

Although active warming devices are effective in intraoperative hypothermia prevention, they constitute a considerable fraction of the total healthcare cost. According to data obtained from device manufacturers, the use of a FAW device can cost 14.55–363.74 USD (100–2500 RMB) per patient per surgery. These expenses include device deployment, consumable parts, and other operating labor fees. To date, few studies have quantitatively compared the cost of these warming devices with the cost caused by IH, as most researchers have focused on the possible harms of hypothermia rather than the economics of using the devices. As a result, the value of investing in warming devices is unclear.

In the present study, we firstly assessed the IH-related cost and then estimated the economic benefits of active warming versus passive warming devices (e.g., cotton blankets or other surface warming devices) using a cost-benefit analysis (CBA) framework with the help of two published meta-analyses. Secondly, by considering the willingness-to-pay (WTP), we investigated the economic value of active warming devices versus passive warming devices for intraoperative hypothermia prevention. Our main goal was to determine the price difference at which active warming is more likely to be adopted over passive warming, providing the price reference for potentially interested parties.

## 2. Methods

### 2.1. Study Design

Analyses were performed from the perspective of healthcare providers because they are concerned with the consequences of IH and the value of warming device choices and are more proactive and guided in the selection of warming devices in clinical practice.

First, we conducted a systematic search of evidence for the safety of intraoperative hypothermia and synthesized this data using a meta-analysis [20]. Then we used data from multiple sources (including literature reports, clinical guidelines, and expert consultations) to determine what clinical treatments would be performed to treat each of the adverse events. The cost of each IH-related adverse event was calculated using the cost of medications (in their minimum dose) and healthcare services across top-grade (Grade 3) hospitals in 19 provinces of China.

Though the above we could estimate: (1) The total cost of IH: based on the incidence of each adverse event multiplied by the cost of such events; (2) The net benefit of active warming devices for IH prevention: using a decision-tree model that compared the differences in IH incidence for active warming versus passive warming devices; (3) The value of active warming devices for IH prevention: discussed by assuming different levels of investment in IH prevention under various levels of WTP.

### 2.2. Incidences of Adverse Events with versus without IH

A meta-analysis we conducted provided us with relevant data [20]. We first conducted a comprehensive database search to identify adverse events associated with intraoperative hypothermia and used these events as keywords. Then, by searching 4 databases: Cochrane Library, PubMed, Clinical Trials, and China National Knowledge Infrastructure (CNKI), we identified studies grouped by hypothermia and reported adverse events within 30 days, which were published prior to February 2019. Finally, nine studies were eventually included in a quantitative analysis [2,3,4,21,22,23,24,25,26] (for more detailed information, please refer to the previously published article [20]).

Compared with patients without IH, those with IH had significantly greater intraoperative blood loss (mean difference (MD) = 131.90 mL, 95% CI: 117.42, 146.38) and incidences of surgical site infection (risk difference (RD) = 0.14, 95% CI: 0.06, 0.21) and intra- or postoperative chill (RD = 0.32, 95% CI: 0.06, 0.58). However, there were no significant differences between patients with versus without IH regarding the duration of surgery (in hours) (MD = −0.16, 95% CI: −0.34, 0.03), hospital stay (in days) (MD = 1.40, 95% CI: −0.35, 3.14), or mortality (RD = 0.00, 95% CI: −0.02, 0.02).

### 2.3. Treatments for Adverse Events and IH-Related Costs

Intraoperative hypothermia-related cost was calculated by aggregating the cost of treating adverse events for IH. Treatments of the adverse events were determined using published studies, clinical guidelines, and expert consultations. The cost for treatment of each adverse event was retrieved from a China-specific national online database. For healthcare services, prices from 28 high-grade (Grade 3) hospitals in 19 provinces were obtained. The 19 included provinces were those included in a national cross-sectional survey of IH conducted in 2014–2015 [27], which the most recent and largest IH studies in China including Beijing, Guangdong, Anhui, Yunnan, Fujian, Gansu, Inner Mongolia, Jiangxi, Tianjin, Hebei, Hainan, Shanghai, Liaoning, Chongqing, Shandong, Jilin, Heilongjiang, Jiangsu, and Hubei. To estimate the cost of medications, the price for each product was estimated based on the anticipated dose and cost per dose. Finally, the cost of treatment for each adverse event was estimated by summing both medications and healthcare utilization for that adverse event, as the following formulae:

Total cost = ∑RD_i_ × unit cost_i_ (for outcomes expressed by count data) (1)

and

Total cost = ∑MD_i_ × unit cost_i_ (for outcomes expressed by continuous data) (2)

where RD_i_ and MD_i_ represent the risk difference and mean difference, respectively.

### 2.4. Cost-Benefit Analysis

**Figure 1:**
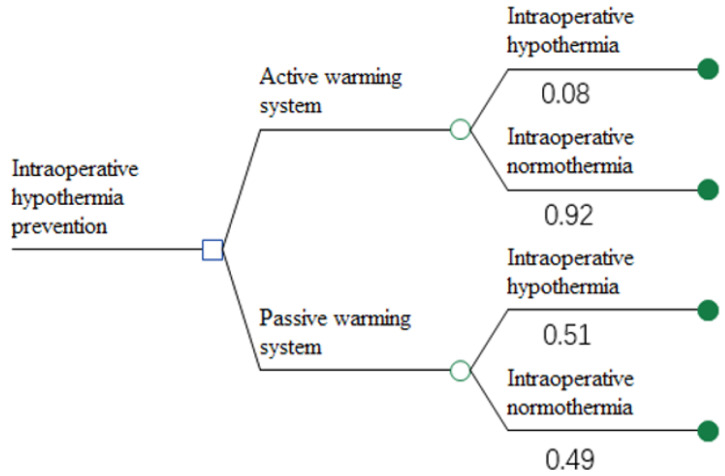
Decision tree for the effect of active versus passive warming on the occurrence of intraoperative hypothermia in major surgeries.

Through the parallel comparison of the decision tree model to compare the value of different warming devices to prevent IH, Figure 1. The incidences of IH under different intraoperative warming devices were determined by a meta-analysis we conducted [28]. As our literature search did not identify any randomized controlled trials of acceptable quality that included patients who received no warming as the control group; therefore, we compared patients managed with active versus passive warming devices.

By searching PubMed, Cochrane Library, Clinical Trials.Gov, and CNKI databases, 8 randomized controlled trial studies published before January 2019 were included. And the risk difference of IH under active versus passive warming was found as −0.42 (95% CI: −0.68, −0.16) (for more detailed information, please refer to the previously published article [28]).

Through the above information, we could calculate △Benefit _active-passive_, which means the cost saved due to the adoption of active rather than passive warming devices to prevent adverse events. Then combined with the difference in the cost of the warming device, △Cost _active-passive_, we could calculate the net benefit of active warming device through the following formula:

Net Benefit = △Benefit _active-passive_ − △Cost _active-passive_ (3)

### 2.5. Uncertainty Analyses

To conduct a comprehensive analysis of different types of uncertainties in our study, a series of uncertainty analyses were performed. Uncertainty in inputs were assessed by sensitivity analysis, uncertainties in methodology were assessed with a scenario analysis.

#### 2.5.1. Deterministic Sensitivity Analysis

Deterministic sensitivity analysis (DSA) were performed to test the robustness of the study results. The following parameters were included: RD of IH under active versus passive warming, differences in the incidences of adverse events in hypothermic versus normothermia patients, and cost of treatment for each adverse event. The variation range of parameters was the maximum and minimum value of each parameter, and the tornado diagram was used to show the influence of different parameter changes on the result.

#### 2.5.2. Probability Sensitivity Analysis

Probability sensitivity analysis (PSA) were performed to test the effect of parameter uncertainty on the study results. It was performed by second-order random sampling (*n* = 2000) the abovementioned parameters, and chances of the dominance of active versus passive warming were then determined under varied WTP. The ranges of parameters were modeled as β (count data) and γ (continuous data) distributions, respectively, the prices of treatments for adverse events were modeled as uniform distributions. Then, a cost-effectiveness acceptability curve (CEAC) was generated to summarize the uncertainty of the cost–benefit analysis and determine the proportions of simulations that were under the WTP thresholds.

In PSA, the net monetary benefit was used as the decision index, and was expressed as

Net monetary benefit = WTP − (△Cost _active-passive_ − △Benefit _active-passive_) (4)

Because △Cost _active-passive_ was decided a priori rather than from empirical studies. In accordance with the price ranges of currently marketed active and passive warming devices, three hypothetical scenarios were analyzed in base-case and sensitivity analysis, where △Cost _active-passive_ was assumed to be 72.70 USD (500 RMB), 145.50 USD (1000 RMB), or 290.10 USD (2000 RMB).

#### 2.5.3. Scenario Analysis

Scenario analysis focuses on the distribution of cost parameters, as the prices of drugs and healthcare services in China were government-regulated rather than random, and the distribution tests indicated that more than half of the cost items followed the normal distribution instead of the γ distribution that is frequently used in cost data fitting. Therefore, in base-case analysis, the prices of treatments for adverse events were modeled as normal distributions, with scenario analysis of uniform distributions for comparison.

All economic analyses were performed with Excel 2016 (Microsoft, Redmond, WA, USA).

## 3. Results

### 3.1. Treatments Costs of Individual Adverse Events

Table 1 summarizes the unit cost and aggregate unit cost per case related to adverse event treatments. The parameters’ range were derived from the maximum and minimum values of reported and published data. All costs were converted to US dollars using the exchange rate on 19 July 2019 (1 USD = 6.8731 RMB). Monetary prices for years other than 2019 were converted to the equivalent prices in 2019, using a discount rate of 5% [29].

### 3.2. Total Cost for the Treatment of Intraoperative Hypothermia

The total treatment cost for one case of IH was calculated by the cost of each item above (Table 1) multiplied by the difference in the incidence of adverse events between IH and without IH (Section 2.2). The formula was as follows and the result was 363.80 USD.

Total cost = RD_surgical site infection_ × related cost + MD_blood loss_ × related cost + RD_chill_ × related cost + MD_surgical duration_ × related cost + MD_hospital stay_ × related cost + RD_mortality_ × related = 0.14 ×243.90 + 1 × 40.40 + 0.32 × 58.80 + −0.16 × 16.00 + 2 × 136.50 + 0 × 1209.10 (5)

### 3.3. Cost-Benefit Analysis of Active Warming Devices versus Passive Warming Devices for Intraoperative Hypothermia Prevention

#### 3.3.1. Base-Case Analysis Result

Since the economic value of preventing one case of IH is equivalent to the cost saved by eliminating its treatment, △benefit _active-passive_ in the decision-tree model was then expressed as follows and the result was 152.80 USD.

△benefit _active-passive_ = hypothermia risk difference of different warming devices × total treatment cost for IH = 0.42 × 363.80 (6)

When the △Cost _active-passive_ was assumed to be 72.70 USD, 145.50 USD, or 290.10 USD, the calculated net benefits were 80.10 USD, 7.30 USD, and −138.20 USD, respectively.

#### 3.3.2. Deterministic Sensitivity Analysis Result

**Figure 2:**
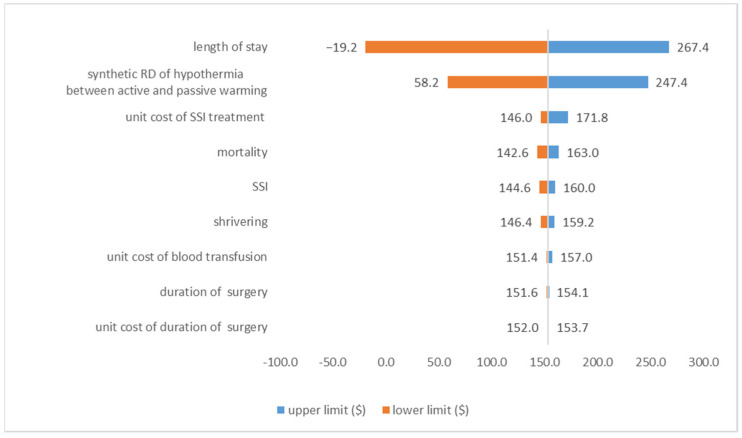
Tornado diagrams showing the sensitivity of △Benefit _active-passive_ to each variable.

The ranges of the parameters were listed in Table 2. As tornado diagram, Figure 2, the △Benefit active-passive was the most sensitive to the duration of hospital stay, followed by synthetic RD of hypothermia between active and passive warming, and unit cost of surgical site infection treatment. Although there was no statistically significant difference between IH or not for the duration of hospital stay, the absolute difference made △Benefit _active-passive_ result reversed. When the duration of hospital stay varied within the 95% CI (−0.35, 3.14), the △Benefit _active-passive_ ranged between −19.20 USD and 267.40 USD. The △Benefit _active-passive_ was insensitive to the duration of surgery and unit cost of the increase in surgical duration, which was related to the small RD and the cheap charge of the duration of surgery.

#### 3.3.3. Probability Sensitivity Analysis Result

After two thousand times Monte Carlo simulation of parameters with a given distribution, the △Benefit _active-passive_ was calculated to be 156.66 USD. That is to say, if the price difference (for active versus passive warming) was <156.66 USD, the active warming was cost-saving. This finding was generally consistent with the finding from the base-case analysis, which declared that the base-case analysis had stability.

**Figure 3:**
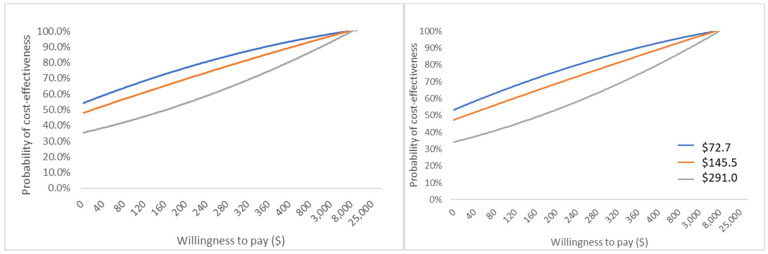
Cost-effectiveness acceptability curves of accepting active warming system. (**Left panel**): normal distribution; (**Right panel**): uniform distribution.

The probabilities of accepting active warming devices are summarized in Table 3. Cost-effectiveness acceptability curves could be seen in Figure 3. Results from CEAC suggested that when the price difference between active and passive warming is 72.70 USD or 145.50 USD and the WTP is zero, the probability of active warming being the preferred option over passive warming was 56.2% or 49.8%, respectively. When the difference was increased to 291.00 USD, the selection of active warming as the preferred option required a minimum WTP of 150.00 USD. This means that active warming is economically feasible only when the decision-maker is willing to pay an additional 150.00 USD or more to avoid one case of IH, which requires the payer to reasonably understand the clinical harm of IH.

#### 3.3.4. Scenario Analysis Result

When uniform distributions of drug and healthcare prices were assumed, the △Benefit _active-passive_ was calculated to be 145.27 USD, so when the price difference (for active versus passive warming) was <145.27, the active warming was cost-saving. This result was similar to that of basic analysis and PSA analysis. CEAC result of scenario analysis was shown in Figure 3, which had the same trend as the normal distribution.

## 4. Discussion

### 4.1. Lower Costs of Intraoperative Hypothermia than Expected

The present study found that the cost associated with IH was only 363.80 USD per case. This differs substantially from another study in which the additional cost of IH was reportedly 2500–7000 USD [8]. This inter-study difference is presumably attributable to differences between countries in prices and provider behavior, therefore that is of a limited reference value. As some studies have described that the costs are usually not transferable between different countries [31].

Our meta-analysis results suggested that IH had lower risks of clinical harm than that reported in individual studies [3,23,32], thus lowering the IH-related cost accordingly. This difference between our meta-analysis and previous individual studies in the IH-related adverse events could partly be explained by the small sample sizes of the individual studies and the earlier time periods in which those studies were conducted, but may also involve other factors, such as the long causal chain from IH to adverse event occurrence and the influence of other clinical practices that may offset the adverse impact of IH. Taking several factors into account, we believed that the results of the systematic review would provide us with more solid information. From the results, considering the lower prices and harm related to IH, we suggest that a healthcare system dominated by public services (e.g., the healthcare system in China) is likely to require considerably lesser resources for IH prevention compared with a healthcare system dominated by private providers.

### 4.2. The Advantage of Performing CBA

Compared with passive warming devices, the biggest advantage of using active warming devices is that they decreased the probability of IH. However, IH is an intermediate effect indicator and cannot be directly used to assess the health benefits of patients, but it may affect the occurrence of intraoperative and postoperative adverse events thereby increasing medical costs. Therefore, we quantified the IH-related additional cost per case using a CBA framework, this method has its unique advantages compared with cost-effectiveness analysis, such as applying to the situation of one cause with more consequences, has multiple outcome indicators and more transparent data, etc., so CBA is more suitable for the evaluation of medical devices.

In CBA, the treatment items for each adverse event were aggregated to give the total cost. This approach contrasts with the method used in other studies in which the cost was determined by calculating the differences in total cost between the group of patients with adverse events versus the group with no adverse events. For example, Wang et al. [33] assessed the cost of infection by separating the patients with postoperative infection from those without and then estimated the difference between the two groups in the total hospitalization fee. To prevent double-counting, we assessed the cost of each event and summed all items by weighted aggregation, with the RD_i_ and MD_i_ calculated in the meta-analysis serving as the weights. This approach used in the present study is more accurate than simple averaging and clearly reveals the contribution of each component, thereby allowing flexible citation and interpretation by different decision-makers (e.g., anesthetists, surgeons, and nurses).

### 4.3. The Complexity of Deciding the Value of Active Warming Devices for IH Prevention

The present analysis suggested that when the price difference between active and passive warming is less than 152.80 USD, active warming is the cost-saving option. When the difference between active and passive warming is increased to 291.00 USD, the WTP must be ≥150.00 USD for active warming to become more cost-effective than passive warming. But the WTP for the benefit of IH prevention is not explicitly stated in any of the literature or clinical guidelines.

Our analysis was performed from the perspective of healthcare providers because patients are typically inadequately aware of the impact of IH. The clinical professionals that are primarily concerned by IH are anesthesiologists, nurses, and clinicians in the fields of orthopedics, gastroenterology, and gynecology. Consultations with anesthesiologists suggested that their WTP ranged from <1000 USD to <10,000 USD. Some anesthesiologists argued that IH prevention is compulsory and that IH should be prevented at all costs. Therefore, even though the WTP varies between clinicians, active warming systems priced at several thousands of US dollars are still acceptable to many clinicians. In contrast, the perspective of the patients may differ from that of the healthcare providers. Patients usually have poor awareness of IH and are concerned with inadequate insurance coverage, and so they perceive the economic advantage of IH prevention as low; thus, the WTP and economic viability of adopting active warming is markedly compromised. These conflicting attitudes between healthcare providers and users highlight conflictive perceptions of the value of the investment in active warming devices.

In the CBA, the risk difference of IH between active and passive devices was −0.42 [28], and the prevention of IH generated a net benefit of only 152.80 USD. Passive warming devices are currently priced at less than 10 USD (i.e., the major cost for cotton blankets is associated with the laundering of the blankets) to 100 USD (e.g., surface warming devices for perioperative body temperature maintenance). In comparison, active warming system costs from tens to thousands of US dollars. When the interventions with active warming devices of different prices are further analyzed, the economic benefits of active warming are expected to decrease in tandem with the reduction in net monetary benefit. Thus, the necessity and affordability of active warming require further in-depth analysis.

### 4.4. Research Novelty and Limitations

Our study demonstrates the economics of warming devices from both clinical and economic indicators. On the one hand, this fills the gap in this field. On the other hand, we got a conclusion that the costs of intraoperative hypothermia are lower than expected, which may provide more reference information for resource allocation and product pricing. And our research contributes to the existing understanding of the value of active warming devices for IH prevention in three ways. First, the characteristics of IH were analyzed based on combined findings from multiple studies, avoiding the bias of individual studies. Second, the study is based on the theory of CBA with a transparent algorithm and traceable raw data. Thus, the methodology is reproducible and could be adapted to other counties. Additionally, we used three levels of WTP in the base-case analysis and discussed the possible impact of conflicting decisions by patients and clinicians on the selection of warming practices.

The present study includes several limitations. First, treatments of some adverse events were estimated from clinical practices, which may involve over- and underestimations of the necessities of certain treatments. For example, patient shivering may suggest an infection, with a workup including blood cultures and attempts to identify a pathogen identification, resulting in potential cost overestimation. Regarding surgical duration, the cost was assumed to only involve additional anesthetic monitoring, which is an underestimation. Overall, the cost assessment was based on the principle of minimum treatment, and the results were likely to have been underestimated overall. A more accurate determination of treatment costs is required. Second, the assessment of the benefits related to active warming devices for IH prevention included only direct benefits, while indirect benefits were ignored (e.g., saving of patient/family working time due to IH prevention). These indirect benefits of IH prevention should be included in further investigations.

## 5. Conclusions

The clinical and economic impact of IH has been widely recognized, and its prevention has been included in clinical guidelines. However, based on our study the harm caused by IH is overestimated, the investment in warming devices should be decided based on an understanding of the risk of IH and WTP of all parties involved. As earlier studies have warned about the harms of IH, many clinicians opt to prevent its occurrence regardless of cost. This attitude may allow further elevation of the price of IH prevention devices. However, under the policy trend of volume-based procurement of medical device consumables in China, manufacturers can only win a broader market if the price is set at a reasonable range, and such price will also be in line with the interests of all parties.

The economic evaluation of our study provides price information for different decision-makers, and we believe that given the fact that in some developing countries the awareness of perioperative body temperature monitoring and hypothermia prevention are not strong [9,34,35,36], the development of more efficient but economical warming devices is warranted.

## Figures and Tables

**Table 1:** Costs (in US dollars) of the treatment of adverse events related to intraoperative hypothermia.

Treatment	Unit Cost Median (min, max)	Aggregate Unit Cost Per Case (min, max)
Surgical site infection		243.9 (128.0, 566.9)
Debridement and suturing	15.6 (9.3, 34.4)	
Daily dressing change	3.1 (1.9, 6.1)	
Antibiotic administration (sulbactam and ampicillin, four times daily for 7 days [30])	19.8 (8.3, 184.2)	
intravenous fluid administration	1.1 (0.5, 2.2)	
Drug susceptibility test	7.4 *^,a^	
Blood culture& pathogen identification	44.6 *^,b^	
Interleukin-6 analysis (three times)	6.5 (3.2, 11.8)	
Procalcitonin analysis (three times)	24.1 (6.4, 45.6)	
Complete blood count test (three times)	3.3 (0.2, 4.5)	
Erythrocyte sedimentation rate analysis (three times)	1.2 (0.6, 1.9)	
Blood transfusion		40.4 (37.1, 50.4)
Cross-matching	0.8 (0.5, 1.9)	
Blood (/200 mL) (whole blood transfusions)	38.0 (35.3, 45.0)	
Blood storage(/bag)	1.6 (1.3, 3.5)	
Chill/Shivering		58.8 (58.8, 58.8)
Tramadol 50 mg	14.3 *^,c^	
Blood culture	44.6 *^,d^	
Hospital stay		136.5 (136.5, 136.5)
Bed use(/day)	136.5 *	
Surgical duration		16.0 (2.5, 27.3)
Monitoring during anesthesia(/h)	16.0 (2.5, 27.3)	
Death		1209.1 (997.6, 2192.4)
Resuscitative care [29]	1209.1 (997.6, 2192.4)	

Data source: Drug costs were determined from acquisition prices released in 2019. Healthcare service costs were determined from the most recent data published by the provinces/municipalities in 2017, except for Inner Mongolia (for which prices published in 2012 were used), Jilin (prices from 2014), Guangdong (prices from 2015), Jiangsu (prices from 2015), Fujian (prices from 2016), Hubei (prices from 2016), Tianjin (prices from 2016), Shanghai (prices from 2016), and Shandong (prices from 2016). * Ranges unlisted for the following reasons: (^a^/^b^) Susceptibility testing and blood culture were conducted based on the suspected microbial species; thus, the test items and unit cost varied substantially between cases. The present study assumed a conservative scenario of one test as the representative case and used the price published in Shanghai as the unit cost (of all provinces/municipalities studied, susceptibility testing and blood culture are priced by the number of experiments/tests performed in Shanghai alone). (^c^) Tramadol was available only as a 100 mg injection in 2019 in the provinces/municipalities studied. (^d^) After the occurrence of chill/shivering, blood culture was required for diagnosis; the price published in Shanghai was used in the analysis, as explained in ^a^/^b^.

**Table 2:** Parameter ranges in the deterministic sensitivity analysis.

Adverse Event	Surgical Site Infection	Intraoperative Blood Loss	Intra/Postoperative Chill	Duration of Surgery	Hospital Stay	Mortality
Difference of adverse events	RD = 0.14 95% CI (0.06, 0.21)	MD = 131.90 95% CI (117.42, 146.38)	RD = 0.32 95% CI (0.06, 0.58)	MD = −0.16 95% CI (−0.34, 0.03)	MD = 1.40 95% CI (−0.35, 3.14)	RD = 0.00 95% CI (−0.02, 0.02)
Cost ($)	243.9 (128.0, 566.9)	40.4 (37.1, 50.4)	58.8 (58.8, 58.8)	16.0 (2.5, 27.3)	136.5 (136.5, 136.5)	1209.1 (997.6, 2192.4)

**Table 3:** Probability of selecting active rather than passive warming under various levels of willingness-to-pay.

△Cost _active-passive_	WTP						
	$0.0	$20.0	$90.0	$150.0	$230.0	$900.0	$2000.0
$72.2 (¥500)	56.2%	57.6%	64.7%	71.9%	80.0%	99.9%	100.0%
$145.5 (¥1000)	49.8%	51.6%	57.5%	62.9%	72.5%	99.5%	100.0%
$290.1 (¥2000)	34.3%	36.7%	44.2%	50.0%	57.2%	98.4%	100.0%

## Data Availability

Data sharing does not apply to this article as no datasets were generated during the current study.

## Abbreviations

CBA	cost-benefit analysis
DSA	deterministic sensitivity analysis
FAW	forced-air warming
IH	intraoperative hypothermia
MD	mean difference
PSA	Probability sensitivity analysis
RD	risk difference
WTP	willingness-to-pay
